# Tell people and lose what I have: Care-seeking, concealment, and disclosure during Sierra Leone’s 2025 clade IIb mpox outbreak

**DOI:** 10.1371/journal.pgph.0007337

**Published:** 2026-09-18

**Authors:** Eric Nzirakaindi Ikoona, Lucy Namulemo, Mary Magdalene Sinnah, Mohamed Alex Vandi, Foday Sahr

**Affiliations:** 1 National Public Health Agency, Freetown, Sierra Leone; 2 Foothills Community-Based Interventions, Monticello, Kentucky, United States of America; 3 Uganda Counselling and Support Services, Kampala, Uganda; Universiti Brunei Darussalam, BRUNEI DARUSSALAM

## Abstract

During Sierra Leone’s 2025 clade IIb mpox outbreak, care-seeking occurred in a social context where formal presentation could expose symptoms, sexualised assumptions, and private relationships to families, workplaces, neighbours, and surveillance systems. We conducted a focused thematic analysis nested within a broader qualitative study across Western Area Urban, Western Area Rural, Bo, and Kenema from 10 March to 28 August 2025. The focused dataset held 70 transcripts. All 42 survivor interviews formed the primary evidence. The remaining 28 were contextual transcripts, drawn from interviews and focus group discussions with healthcare workers, contact tracers, and community members. Analysis used the Framework Method, dual coding, reflexive memos, matrix charting, and member checking with survivor participants. We identified four linked processes: protective misrecognition through socially safer diagnostic categories; disclosure risk as the point where stigma, cost, distance, relationship risk, and service trust converged; health system co-production of delay through early diagnostic uncertainty, referral gaps, and limited diagnostic access; and enabling conditions that made care-seeking safer, especially trusted intermediaries and visible confidentiality practices. Twenty-eight of the 42 survivor accounts met the criteria for pathway classification: 16 supported disclosure pathways and 12 forced concealment pathways, including three transitional cases that shifted after a trusted intermediary emerged. The other 14 survivor accounts lacked one or more pathway criteria and contributed contextual survivor evidence. Gender, intimate partnership risk, contact tracing visibility, and memories of Ebola and COVID-19 shaped disclosure decisions. The framework specifies how disclosure operates as a convergence point in high-stigma outbreak care-seeking. Outbreak preparedness should design confidentiality, intermediary, and contact-tracing systems that make disclosure safe enough to perform.

## Introduction

Infectious disease outbreak research consistently shows that people delay formal care because stigma, fear, cost, distance, distrust, and service inaccessibility interact rather than act as isolated barriers [1,2]. This paper extends that literature by examining where interacting pressures converge within lived care-seeking sequences.

A large disclosure literature, especially from HIV and other concealable stigmatised conditions, shows that disclosure decisions involve social risk, anticipated reaction, relationship consequences, and control over information [3–5]. Stigma theory and the Health Stigma and Discrimination Framework also show that anticipated, enacted, internalised, and associative stigma shape health behaviour across multiple levels [6,7]. The contribution lies in specifying how disclosure operates during a visible, acute, sexually moralised outbreak, where formal care-seeking can transform private illness into public identity.

We use the term disclosure model of care-seeking to describe a mid-range framework in which formal presentation to services represents the point at which a person must allow others to know, observe, or infer their illness status, causing stigma, cost, relationship risk, trust, and service barriers to converge. Distance then measures more than travel time, because every journey adds an occasion to be seen. Cost extends beyond money to work, housing, partnership, and reputation. Patients also judge service quality by whether a facility protects privacy. Barrier reduction remains necessary, and it achieves little while the act of disclosure feels unsafe.

Sierra Leone’s 2025 clade IIb mpox outbreak provides a high-stakes setting for examining this process. Mpox was declared a public health emergency in Sierra Leone in January 2025, and by June 2025 the country reported more than 4,000 confirmed cases [8–10]. Clinical and epidemiological reports from the outbreak described frequent genital involvement and a pattern consistent with sustained human-to-human transmission [11,12]. These features gave formal care-seeking particular social meaning because genital symptoms and perceived sexual transmission could be read through assumptions about sexual behaviour, infidelity, and moral blame.

This paper addresses three questions: how did disclosure risk organise care-seeking decisions during Sierra Leone’s mpox outbreak; what distinguishes supported disclosure from forced concealment pathways; and what enabling conditions shifted trajectories toward earlier and safer care-seeking? We also examine how gender, intimate partner risk, contact tracing, and memories of Ebola and COVID-19 shaped these pathways.

## Methods

### Study design and relationship to the parent study

This paper reports a focused secondary thematic analysis of transcripts generated by a larger multi-perspective qualitative study of Sierra Leone’s 2025 clade IIb mpox outbreak, published in PLOS Global Public Health [13]. The parent study examined outbreak experiences across four stakeholder groups, namely mpox survivors, healthcare workers, community members, and contact tracers, in Western Area Urban, Western Area Rural, Bo, and Kenema between 10 March and 28 August 2025. Its full design, setting, sampling, recruitment, consent procedures, interview and focus group conduct, languages, transcription, translation, training, and analytic infrastructure are reported in the parent paper [13]. The present analysis used de-identified transcripts collected under the same approved parent protocol; no new data collection occurred. The present analysis draws on the same empirical corpus but addresses a narrower conceptual question, namely how disclosure risk organised care-seeking, concealment, and presentation to services. We followed the Framework Method [14,15] and reported the study in line with COREQ [16] (S1 Table).

### Focused dataset and source selection

The parent corpus comprised 94 individual semi-structured interviews (SSI) and 12 focus group discussions (FGD). For this analysis we constructed a focused dataset of 70 transcripts: all 42 survivor interviews and 28 contextual transcripts purposively selected because they directly addressed care-seeking, disclosure, diagnostic pathways, contact tracing, or the social consequences of being identified as a case or contact. The 28 contextual transcripts comprised 16 individual interviews and all 12 focus groups: 8 healthcare worker interviews and 4 healthcare worker groups, 6 contact tracer interviews and 2 contact tracer groups, and 2 community interviews and 6 community groups.

We treated survivor interviews as first-person care-seeking narratives and treated healthcare worker, contact tracer, and community accounts as contextual, observational, or normative evidence. Fourteen survivor accounts that lacked sufficient pathway criteria were also retained as contextual survivor evidence and contributed to interpretation of the four process findings but were not classified into pathways. No survivor focus groups were held in the parent study, because illness narratives, genital symptoms, suspected sexual exposure, and family or workplace disclosure were too sensitive for a group setting. Individual interviews therefore carried the experiential analysis, while focus groups informed collective meaning, operational practice, and points of agreement or disagreement across roles. We did not treat focus groups as confirmation of interviews. We compared epistemic positions across sources in shared matrices, so that convergence strengthened credibility and divergence prompted further interrogation.

Focus group discussions contained an average of 12 participants and lasted a mean of 87 minutes, with a range of 72–110 minutes. Groups were organised by stakeholder type and district so that participants discussed experiences with peers who occupied similar roles. The mix of participants was informed by feasibility, cadre, district representation, and the need to avoid placing survivors in group settings where illness history, genital symptoms, sexualised assumptions, or family and workplace disclosure could be exposed. Facilitators used a semi-structured guide, began with confidentiality ground rules, invited quieter participants to contribute, encouraged disagreement, and avoided prompts that required disclosure of identifiable patients, colleagues, partners, or contacts. Where group discussion suggested that a sensitive issue needed private exploration, facilitators offered follow-up individual interviews.

### Data collection instruments

Separate interview and focus group guides were developed for each stakeholder group in the parent study. They drew on the Health Stigma and Discrimination Framework, the Social Ecological Model, the outbreak care-seeking literature, and the HIV disclosure literature [3–7], were piloted with five individuals per group, and were refined with input from Community Health Officers, District Health Management Team staff, treatment centre staff, and pilot participants. No formal community advisory board co-designed the study. The guides relevant to this analysis are provided as S1 Appendix.

### Language, transcription, translation, and quotation presentation

Interviews and focus groups were conducted in Krio, Mende, Temne, or English according to participant preference [13]. Sessions were audio-recorded with consent, transcribed verbatim in the original language, and translated into English by bilingual team members. Randomly selected segments from 20% of transcripts were back-translated to check consistency of meaning. This check identified minor wording differences but no discrepancies that altered the themes. Quotations were lightly edited for readability and removal of identifying details without altering substantive meaning. Each quotation identifies the data source as SSI or FGD, followed by participant role, gender, age where available, and district. Role codes are survivor (S), healthcare worker (HCW), contact tracer (CT), and community member (CM). District codes are Western Area Urban (WAU), Western Area Rural (WAR), Bo (BO), and Kenema (KEN). Healthcare worker labels add the cadre at the end. A quotation labelled SSI, S-M-31-WAU therefore comes from an individual interview with a male survivor aged 31 in Western Area Urban.

### Analysis

Analysis followed the Framework Method through familiarisation, codebook development, indexing, matrix charting, cross-case comparison, and interpretive synthesis [14,15]. ENI and LN independently coded all 70 transcripts using a co-developed codebook and resolved differences by consensus. NVivo 14 supported coding and matrix charting. Reflexive memos documented analytic decisions, disagreement resolution, the principal investigator’s insider position, and emerging interpretations. Focus group transcripts were coded with the same process codes as interviews but were flagged as group-level discourse. The coding framework is provided as S2 Table.

Pathway classification used three criteria: the account described a trajectory from illness onset to formal care-seeking or continued non-seeking; the account referred explicitly to the social dimensions of disclosure risk; and the account gave sufficient detail on the presence or absence of a trusted intermediary. Twenty-eight of 42 survivor accounts met all three criteria and were assigned to a pathway. The remaining 14 survivor accounts were retained as contextual survivor evidence. They lacked one or more criteria, generally because they centred on symptom interpretation, recovery, post-diagnosis stigma, or access barriers without enough detail on the full onset-to-care trajectory or intermediary presence.

Because this was a focused secondary analysis of an existing corpus, we did not set out to achieve de novo thematic saturation. We instead assessed analytic sufficiency, namely whether the focused dataset held enough information power to develop and refine the four care-seeking processes and the pathway typology [17]. Survivor transcripts were coded in the chronological order in which the parent study collected them. The codebook and the three pathway criteria were established on the first 25 survivor transcripts. Coding of the remaining 17 survivor transcripts produced no new process codes and no new pathway features, which indicated informational sufficiency for the analytic question. Subgroup numbers were too small to support saturation within gender, age, or district strata, so we present subgroup observations as analytic patterns rather than saturated categories.

### Rigour, transferability, and member checking

Credibility was supported by dual coding, matrix comparison across participant groups, and explicit differentiation between lived, professional, and community accounts. Dependability and confirmability were supported by a codebook, an audit trail, and reflexive memos. Transferability was addressed through thick description of context, participants, outbreak setting, and pathway criteria, and through stated scope conditions rather than statistical generalisation [18]. The disclosure model is presented as most applicable where diagnosis carries high social stakes, where symptoms or care-seeking are visible, and where confidentiality systems are weak, mistrusted, or socially observable. Member checking for this analysis was conducted with five survivor participants drawn from the parent study’s member-checking process. They reviewed summaries of the supported disclosure, forced concealment, and transitional pathways, confirmed that the typology reflected their experience, and emphasised that confidentiality and trusted accompaniment should be foregrounded, which we incorporated into the pathway definitions and implications.

### Positionality and reflexivity

This analysis was led from within Sierra Leone’s public health system and anchored in the National Public Health Agency (NPHA). ENI, the principal investigator, is a physician and public health researcher and Strategic Advisor at the NPHA. FS, the co-principal investigator, is the Executive Director of the NPHA. MAV and MMS are co-investigators and Sierra Leonean public health professionals at the NPHA with direct roles in the national outbreak response and surveillance system. LN is a doctorate-trained social scientist and mental health researcher with qualitative experience in HIV and infectious disease research in East and West Africa, and had no role in the Sierra Leone mpox response.

The investigators’ positions as public health practitioners and as Sierra Leoneans living through the same outbreak shaped data collection and interpretation. This insider position aided contextual understanding but could also influence how participants responded and how their accounts were read. To reduce this risk, most interviews and focus groups were conducted by trained research assistants recruited from outside the national response coordination structure, and participants were told that participation was voluntary and would not affect care, services, surveillance follow-up, or employment. LN provided an external analytic counterweight during coding and interpretation, and reflexive memos recorded where insider assumptions required questioning or re-analysis.

### Ethics

The parent study was approved by the Sierra Leone Ethics and Scientific Review Committee (SLESRC-2025-049) on 28 February 2025, and this secondary analysis falls within that approval. All participants gave written informed consent, with witnessed thumbprint consent for those who could not write. Identifiers were removed during transcription, and quotations were altered where necessary to prevent recognition. Because this manuscript analyses existing de-identified transcripts collected under the approved parent protocol, no new participant contact, consent, or data collection was required for the secondary analysis.

## Results

Four interconnected processes shaped care-seeking trajectories: illness recognition through socially safer diagnostic categories; disclosure risk as the organiser of care-seeking decisions; health system co-production of delay; and enabling conditions for timely care. These processes produced two pathway types, supported disclosure and forced concealment, together with transitional cases and survivor accounts that could not be classified.

### Process 1: Illness recognition through safer diagnostic categories

The first stage of care-seeking was seldom immediate recognition of mpox. Survivors described interpreting symptoms through less stigmatising categories such as chickenpox, allergy, malaria, scabies, or a sexually transmitted infection that felt more manageable than mpox. This was sometimes genuine uncertainty and sometimes protective misrecognition: a way to delay the social implications of naming mpox.

“I knew it was not ordinary chickenpox because I am too old and the sores looked different. But I told myself it was chickenpox because as long as I was telling myself that, I did not have to think about the other possibility and what it would mean for my life.” (SSI, S-M-31-WAU)

“The first week I thought it was something I had eaten. The second week I thought maybe it was an allergy. I was not stupid. I knew. But knowing and accepting that you know are different things when accepting means your whole situation changes overnight.” (SSI, S-F-28-WAR)

Healthcare workers also described this pattern at first presentation. They interpreted it as a mixture of low early awareness, symptom overlap with common rash illnesses, and fear of the social meaning attached to mpox.

“We saw patients who had been treating themselves for chickenpox for ten days before they came to us. Some had been to pharmacies and bought medications. They were not ignoring their illness. They were treating it as something safer.” (SSI, HCW-F-33-WAU-Nurse)

### Process 2: Disclosure risk as the point where barriers converged

By the time many survivors delayed care, they already recognised that symptoms were serious. The central calculation was whether the social consequences of formal care-seeking were survivable. Formal care-seeking changed who could know, infer, and act on that illness. This calculation brought stigma, livelihood, relationship risk, distance, and facility trust together at one threshold.

“I knew something was very wrong. But going to the hospital meant people would see me going in. In my area news travels fast. I kept saying to myself, another day, another day, and maybe it will pass before anyone has to know.” (SSI, S-M-22-WAU)

“I know two people in my area who got mpox before me. I saw what happened to both of them. One lost his job. The other one, his wife left him and took the children to her parents. When I got sick, I already knew what my options were. Tell people and lose what I have, or say nothing and try to manage on my own.” (SSI, S-M-31-WAR)

The second account also shows why disclosure risk was gendered and relational. A diagnosis associated with genital lesions could be interpreted as evidence of sexual behaviour outside a relationship. For some men, the feared consequence was accusation of infidelity, partner separation, or loss of children. For some women, the feared consequence included moral judgement, rejection, or violence linked to assumptions about sexual exposure. Gender shaped what disclosure could cost rather than dividing survivors along a simple male-female line.

Disclosure risk required active concealment labour. One urban participant described maintaining multiple explanations at once while symptoms worsened:

“I wore long sleeves even though it was hot. I kept the collar high. I told my employer I had a family emergency and was working from home. I told my landlord I had flu so he would not come to fix things in the flat. I told my girlfriend I was visiting my mother. I kept all of this up for eight days.” (SSI, S-M-31-WAU)

Distance and cost carried social weight beyond logistics. In rural and peri-urban areas, travel multiplied disclosure events because the journey, waiting area, referral process, and repeated facility visits exposed symptoms to more observers.

“From my village to the hospital in Kenema is more than two hours by motorbike. I was sick, I was in pain, and I had to borrow money for the journey. When I got there, they told me to wait. I sat in a waiting area with other people for three hours. Everyone could see the sores on my face. I said to myself, I will never come here again if I can avoid it.” (SSI, S-M-36-KEN)

“I walked for forty minutes to the community health centre. When I got there, the nurse looked at me and said she was not sure what it was and I should go to Kenema. I did not have money for Kenema that day. I went home. I came back three days later with the money. By then the sores were worse and more visible.” (SSI, S-F-34-BO)

Disclosure was not the main driver of delay in every account. Some survivors emphasised transport cost, physical weakness, need to keep earning daily income, or confusion caused by inconsistent early diagnoses. However, even in these accounts, the social meaning of being seen with mpox often intensified the practical barrier.

### Process 3: Health system co-production of delay and disclosure

Community reluctance was one source of delay among several. Survivors who crossed the disclosure threshold often encountered misdiagnosis, unclear referral pathways, or delayed laboratory confirmation. These system factors mattered ethically because people who took the risk of seeking care did not always receive timely diagnosis or confidential management.

“I went to the clinic near my house and the nurse told me it was a bad skin reaction and gave me cream. A week later when the sores were spreading, I went back, and a different nurse said it might be something sexually transmitted and started me on tablets. Only at the bigger hospital did someone say the word mpox and send for a swab.” (SSI, S-M-29-WAU)

“We were seeing patients who had already been to two or three facilities before us. They had already been treated for chickenpox, for scabies, sometimes for syphilis. By the time they reached the treatment centre, some of them had been sick for two weeks. Part of that is community delay. But part of it is us. The system was not ready for this disease at the peripheral level.” (SSI, HCW-M-44-WAU-Physician)

Healthcare workers described overlapping presentations with chickenpox, scabies, syphilis, and allergic rashes, alongside limited tools to confirm mpox quickly. Healthcare worker accounts did not contradict survivor accounts of why delay occurred; they described the same encounters from the system side and differed mainly in emphasising health system readiness over individual behaviour.

Contact tracing was another site where disclosure could be protected or amplified. Contact tracers could function as trusted intermediaries when they approached people discreetly, explained confidentiality, and helped cases think through whom to tell. Yet tracing could also become a forced disclosure event when visits occurred at workplaces, doorways, or visibly in neighbourhoods.

“Many patients are reluctant to name their contacts, especially if the contact was through sexual activity. They are afraid of what will happen to those people, or they do not want to reveal their private behaviour.” (SSI, CT-M-34-WAU)

“My neighbour was identified through contact tracing. The whole street knew before she even knew herself. People saw the contact tracers going to her house. By the time the result came back, everyone had already decided she had mpox and had already started avoiding her. She told me she felt like she had been stripped in public.” (FGD, CM-F-WAU)

“I was identified as a contact. The contact tracer came to my workplace. My colleagues saw. By the time I was tested and the result was negative, the damage was already done. People at work already knew I had been in contact with mpox. The negative result did not change what they thought about me.” (SSI, CM-M-34-WAU)

Contact tracing therefore operated as disclosure work. Tracing collects names and changes who knows, when they know, and what they infer. This was heightened in Sierra Leone because communities carried memories of Ebola and COVID-19 response practices, where public health visits, isolation, ambulance movement, and visible surveillance could signal infection before anyone spoke.

### Process 4: Enabling conditions for timely care

Three conditions helped survivors seek care earlier: practical communication that connected symptoms to an action point; a trusted intermediary; and visible confidentiality at the point of care. These conditions did not remove stigma from the community, but they redistributed disclosure risk enough for some people to act.

“There was a health message I heard about mpox that described the sores exactly. When I saw the first one appear, I did not wait. I went the same day because I already knew what it was and where to go.” (SSI, S-M-33-WAU)

“My cousin is a nurse. When I got sick, I called her first. She told me what to expect and she went with me to the hospital and spoke to the staff there. I think without her I would have been treated differently. Having someone who knows how these things work made it possible to go.” (SSI, S-F-26-WAU)

“The community health worker in my area had spoken to us about mpox at a community meeting. She had explained what the sores looked like and said that if we had something like that we should come to her first and she would help us. When I got sick, I went to her. She took me to the health centre herself.” (SSI, S-F-24-WAR)

“My friend came with me. She held my hand when they took the swab. When the nurse spoke to me in a way I did not understand, my friend explained it. When I was afraid they would tell people, she spoke to the nurse and asked about confidentiality. I could not have asked that myself.” (SSI, S-F-22-WAU)

### Pathway typology: Supported disclosure, forced concealment, and transitional cases

Cross-case comparison identified two primary pathway types. Of the 42 survivor accounts, 28 met all typology criteria: 16 supported disclosure pathways and 12 forced concealment pathways. The forced concealment group included three transitional cases in which concealment shifted to supported disclosure after a trusted intermediary emerged mid-trajectory. These transitional cases were retained within the forced concealment count because the primary trajectory began as concealment, but we analyse them separately because they demonstrate pathway modifiability. Table 1 summarises the defining conditions, trajectories, typical timing, social consequences, and response implications of each pathway.

**Table 1:** Supported disclosure, forced concealment, and transitional care-seeking pathways.

Feature	Supported disclosure pathway	Forced concealment pathway	Transitional cases
**Case count**	n = 16	n = 12	n = 3, included within the n = 12 forced concealment pathways because each began with concealment before shifting to supported disclosure.
**Defining condition**	A trusted person absorbed part of the social cost of disclosure before or during presentation to services.	No trusted disclosure relationship was available; concealment continued until it became physically or socially impossible.	A trusted intermediary appeared after concealment had already begun, changing the trajectory.
**Trajectory**	Private disclosure to trusted person - > supported presentation - > some control over information spread.	Progressive concealment - > involuntary identification or forced presentation - > reduced control over information spread.	Initial concealment - > intermediary identification - > supported presentation or safer disclosure.
**Typical timing**	Usually within the first week of illness.	Usually second week or later, often triggered by an external event.	Care-seeking followed an initial period of concealment and the emergence of a trusted intermediary.
**Social consequence**	Moderated by accompaniment, advocacy, and confidentiality negotiation.	More severe because being identified compounded disease stigma with the stigma of hiding.	Consequences depended on how quickly the intermediary reduced public exposure and helped manage disclosure.
**Response implication**	Build trusted intermediary networks before outbreaks and make confidentiality visible at entry points.	Reduce public exposure during care-seeking and contact tracing; protect people from avoidable identification.	Design interventions that can move people from concealment to supported disclosure even after delay begins.

Timing categories are based on survivor-reported illness onset and care-seeking trajectories described during individual interviews. n = number of survivor accounts assigned to the pathway; denominator is the 28 of 42 survivor accounts that met all three pathway criteria.

The 14 unclassified survivor accounts lacked enough detail for pathway assignment, and none contradicted the typology. Some described symptoms and eventual treatment but not the social decision process; some focused on stigma after diagnosis; some described transport or cost barriers without enough information about intermediary support; and some centred recovery rather than the onset-to-care trajectory. We therefore used them to refine the four process findings but did not count them as pathway cases.

The transitional cases show that forced concealment can change. In each one, a person who was initially managing symptoms alone moved toward care after identifying someone who could hold information, interpret symptoms, provide transport or accompaniment, and help negotiate confidentiality. The shift began when a credible person received the first disclosure, protected the information, and helped negotiate the next step.

### Gendered and relational dimensions of disclosure

Gender shaped the expected cost of disclosure rather than its intensity. For many male survivors, the feared consequence was that genital symptoms would be read as proof of sexual behaviour outside the relationship, threatening marriage, children, standing, and income.

“When the sores started, my first worry was not the hospital. It was my wife. Things were already hard between us, and I knew that if this came out, she would believe I had been with other women. A disease like this, in that place on the body, there is only one story people tell about it. I would lose my marriage and my children before I ever lost my job.” (SSI, S-M-33-WAU)

For many female survivors, the feared consequence was moral judgement of their character, rejection by family, or the risk of violence once symptoms were interpreted as evidence of unacceptable sexual conduct.

“For a woman it is not the same. If people see you have this, they do not say you are sick. They ask what kind of woman you must be. My biggest fear was not the pain. It was that my brothers would hear, and what they would say, and what they might do. I would rather have suffered quietly than have my own family look at me that way.” (SSI, S-F-27-BO)

These patterns were not uniform. Economic vulnerability and relationship risk cut across gender, and younger participants additionally feared peer disclosure and exposure through social media. Disclosure therefore attached to different social risks across different social positions, and those risks shaped what a trusted intermediary needed to protect.

## Discussion

### Principal findings

This focused analysis shows that care-seeking during Sierra Leone’s 2025 mpox outbreak was often a disclosure-mediated process. People weighed the clinical benefit of care against the social consequences of becoming identifiable as a person with a stigmatised, visible, and sexually moralised condition. Four processes produced delay or timely care: protective misrecognition, disclosure risk convergence, health system co-production of delay, and enabling conditions such as trusted intermediaries and visible confidentiality.

### Contribution relative to existing disclosure literature

Disclosure processes, HIV status disclosure, stigma management, and the social consequences of revealing a concealable identity are well established in the literature [3–5,19–21]. This paper adds an outbreak-specific account of how disclosure operates when illness is acute, visible, subject to surveillance, and linked to sexualised assumptions. In this setting, disclosure is the point at which multiple barriers reinforce one another.

The supported disclosure versus forced concealment typology adds an operational tool. It describes the social structure of the pathway, namely whether a trusted person is available to absorb part of the disclosure cost before formal care. This distinction can guide intervention design, contact tracing practice, and evaluation of confidentiality systems.

### From barrier interaction to mechanism of convergence

Established care-seeking and outbreak response frameworks already recognise that barriers interact rather than acting in isolation [1,2,22]. The disclosure model locates where that interaction concentrates. Cost, distance, stigma, trust, and service quality converged at the point where a person had to allow someone else to know or infer their illness. This helps explain why reducing one barrier may not shift behaviour if disclosure itself remains unsafe.

### Epidemic memory, contact tracing, and non-disclosure

Ebola and COVID-19 shaped disclosure in Sierra Leone. Previous outbreaks created powerful community memories of public health teams entering homes, isolation being socially visible, ambulances attracting attention, and survivors or contacts being marked by association [23–25]. These memories created expectations about what happens socially when outbreak systems identify a person or household. In the mpox outbreak, those memories interacted with the sexualised stigma of clade IIb to make non-disclosure a rational protective strategy for some participants.

Contact tracers therefore occupied an ambivalent role. They could reduce stigma by offering confidential, trusted, explanatory support. They could also amplify stigma if tracing visits, workplace notifications, or public household approaches made exposure visible. Trusted social ties may also shape health behaviour by distributing practical and social risk [26]. Ethical contact tracing in high-stigma settings must protect confidentiality in records and in the choreography of field practice: how teams dress, where they speak, when they visit, and how they avoid marking households or workplaces [27,28].

### Rare outbreak diagnosis and health system readiness

Junior health workers cannot be expected to recognise a rare outbreak presentation without diagnostic support. Early mpox recognition was difficult because rash illnesses are common, diagnostic tools were limited at peripheral level, and outbreak knowledge developed as the epidemic evolved. The appropriate expectation is readiness for differential diagnosis, red-flag recognition, safe referral, and escalation when symptoms do not fit routine categories. This reframing aligns with healthcare worker evidence from the multi-country mpox response and global reviews of health worker knowledge during early outbreaks [29,30].

### Gender, intimate partnerships, and supported disclosure

Supported disclosure was especially complex where one partner showed symptoms and the other did not. In those situations, the illness could be interpreted as evidence of infidelity or socially unacceptable sexual behaviour, even when the actual transmission route was unknown. This helps explain why a trusted intermediary mattered. The intermediary helped survivors manage the relational risk of naming mpox, contacting partners, and entering care without losing all control over information.

### Implications for outbreak response

Three implications follow. First, confidentiality should be designed as a visible system property. Patients need to see privacy in triage spaces, waiting areas, referral pathways, contact tracing visits, and staff behaviour. Second, trusted intermediary networks should be prepared before cases accelerate. Community health workers, survivor advocates, peer supporters, and trusted clinicians can act as first disclosure points who help people move from concealment to care. Third, contact tracing should include stigma-safety protocols that prevent field practice from becoming public identification.

These measures belong inside the technical response. In a high-stigma outbreak, they shape whether people present early enough for clinical care, isolation, testing, vaccination, and contact tracing to work. The transitional cases suggest that even after concealment begins, trusted intermediary activation can redirect the pathway.

### Transferability and scope conditions

The disclosure model is offered as a mid-range conceptual framework rather than a universal theory [31]. Its explanatory force is likely strongest where three conditions hold: diagnosis carries high social stakes, symptoms or care-seeking are visible, and confidentiality systems are mistrusted or socially observable. Tuberculosis may share these conditions because diagnosis can involve prolonged uncertainty, stigma, and social consequences of being identified as infectious. HIV testing and care share disclosure dynamics because individuals often weigh anticipated social consequences before revealing health information. Mpox among men who have sex with men provides another example where visible symptoms and sexualised interpretations can influence disclosure decisions. Some mental health presentations may share similar mechanisms when seeking care risks social judgement or altered identity within family and community networks [19,22,32–35]. Where stigma is low and confidentiality is trusted, disclosure may not organise care-seeking in the same way.

### Limitations

Survivors who never entered formal care are not represented, and their trajectories may include more extreme concealment. The cross-sectional design limits reconstruction of changing decisions over time. Social desirability may have shaped accounts of sexual exposure, timing of care-seeking, and contact naming. The typology was assigned to 28 of 42 survivors, not the full survivor sample, because we required sufficient detail on trajectory, disclosure risk, and intermediary presence. The 14 unclassified survivor accounts contributed contextual survivor evidence and are not pathway cases. Gendered patterns were exploratory because subgroup sizes were small. Finally, the PI’s public health role could have shaped participant responses, although fieldwork structure, external co-analysis, reflexive memoing, and voluntary consent procedures were used to reduce this risk.

## Conclusions

Care-seeking during Sierra Leone’s 2025 clade IIb mpox outbreak was shaped by the social structure of disclosure. Existing barrier models remain important. Stigma, cost, distance, relationship risk, trust, diagnostic uncertainty, and contact tracing visibility converged at the moment when private illness risked becoming public identity. Supported disclosure pathways occurred when a trusted person absorbed part of that social cost. Forced concealment occurred when no such support was available and disclosure became involuntary. Transitional cases show that pathways can change when intermediary support appears. Outbreak preparedness should therefore build disclosure-safe infrastructure: trusted intermediary networks, visibly confidential services, stigma-safe contact tracing, and communication that offers low-exposure routes into care. In high-stigma outbreaks, making disclosure safe enough to perform is a prerequisite for timely diagnosis, surveillance, and response.

## Supporting information

S1 Table COREQ checklist for this focused qualitative analysis. Completed according to Tong et al. [16].
(DOCX)

S2 Table Analytic coding framework used for this focused analysis, including pathway typology criteria, process codes, contextual evidence rules, coding decisions, and disagreement resolution. (DOCX)

S1 Appendix Semi-structured interview and focus group discussion guides used to collect survivor, healthcare worker, contact tracer, and community member data for the parent study and this focused analysis. (DOCX)
