# Supplementary material for: Tell people and lose what I have: Care-seeking, concealment, and disclosure during Sierra Leone’s 2025 clade IIb mpox outbreak
Source: PLOS Glob Public Health. 2026 Sep 18;6(9):e0007337. doi: 10.1371/journal.pgph.0007337 (PMC13588523; doi:10.1371/journal.pgph.0007337)
Supplement: Supplementary file 1 [file pgph.0007337.s001.docx]

**Supporting Information**

Tell people and lose what I have: care-seeking, concealment, and disclosure during Sierra Leone’s 2025 clade IIb mpox outbreak

Eric Nzirakaindi Ikoona, Lucy Namulemo, Mary Magdalene Sinnah, Mohamed Alex Vandi, Foday Sahr

**S1 Table. COREQ checklist for this focused qualitative analysis**

This checklist applies to the focused secondary thematic analysis reported in this manuscript. Full parent-study procedures are reported in the accepted parent publication and referenced in the manuscript.

| **Item** | **COREQ guide question / description** | **Where addressed** |
| --- | --- | --- |
| 1 | Interviewer/facilitator | Methods: data collection, positionality and reflexivity |
| 2 | Researcher credentials | Methods: positionality and reflexivity; parent study reference |
| 3 | Researcher occupation | Methods: positionality and reflexivity |
| 4 | Researcher gender | Parent study methods; research team records |
| 5 | Researcher experience and training | Parent study methods; data collection instruments |
| 6 | Relationship established before study commencement | Methods: recruitment and consent described in parent study; no pre-existing research relationship required |
| 7 | Participant knowledge of interviewer | Methods: ethics, consent, and positionality sections |
| 8 | Interviewer characteristics reported | Methods: positionality and reflexivity |
| 9 | Methodological orientation and theory | Methods: study design; Introduction; Framework Method and disclosure model |
| 10 | Sampling | Methods: focused dataset and source selection |
| 11 | Method of approach | Parent study methods; focused dataset section |
| 12 | Sample size | Methods: focused dataset and source selection; Results typology section |
| 13 | Non-participation | Parent study methods; not separately assessed for this secondary analysis |
| 14 | Setting of data collection | Methods: study design and parent study description |
| 15 | Presence of non-participants | Parent study data collection procedures; confidentiality ground rules |
| 16 | Description of sample | Methods: focused dataset and source selection |
| 17 | Interview guide | S1 Appendix |
| 18 | Repeat interviews | No repeat interviews were conducted for this focused secondary analysis |
| 19 | Audio/visual recording | Methods: language, transcription, translation, and quotation presentation |
| 20 | Field notes | Parent study methods; reflexive memoing described in this manuscript |
| 21 | Duration | Methods: focus group conduct paragraph; parent study methods |
| 22 | Data saturation | Methods: analysis; analytic sufficiency statement |
| 23 | Transcripts returned | Not returned individually; member checking used summary typology feedback |
| 24 | Number of data coders | Methods: analysis |
| 25 | Description of coding tree | S2 Table |
| 26 | Derivation of themes | Methods: analysis; Results process and typology sections |
| 27 | Software | Methods: analysis |
| 28 | Participant checking | Methods: rigour, transferability, and member checking |
| 29 | Participant quotations presented | Results |
| 30 | Data and findings consistent | Results and Discussion; matrix comparison across sources |
| 31 | Clarity of major themes | Results: four processes and pathway typology |
| 32 | Clarity of minor themes and diverse cases | Results: unclassified accounts, transitional cases, gendered and relational dimensions |
