# Supplementary material for: Tell people and lose what I have: Care-seeking, concealment, and disclosure during Sierra Leone’s 2025 clade IIb mpox outbreak
Source: PLOS Glob Public Health. 2026 Sep 18;6(9):e0007337. doi: 10.1371/journal.pgph.0007337 (PMC13588523; doi:10.1371/journal.pgph.0007337)
Supplement: Supplementary file 2 [file pgph.0007337.s002.docx]

**Supporting Information**

Tell people and lose what I have: care-seeking, concealment, and disclosure during Sierra Leone’s 2025 clade IIb mpox outbreak

Eric Nzirakaindi Ikoona, Lucy Namulemo, Mary Magdalene Sinnah, Mohamed Alex Vandi, Foday Sahr

**S2 Table. Analytic coding framework used for this focused analysis**

This table summarises process codes, pathway typology criteria, pathway categories, contextual evidence rules, and coding boundaries used in the focused secondary analysis. The ten process codes map onto the four processes reported in the Results. Protective misrecognition corresponds to Process 1. Disclosure risk convergence, concealment labour, and distance as exposure multiplier correspond to Process 2. Health system co-production of delay, differential diagnosis and red-flag recognition, and contact tracing as disclosure work correspond to Process 3. Trusted intermediary, visible confidentiality, and actionable communication correspond to Process 4. ENI and LN independently coded all 70 transcripts and resolved differences by consensus. Reflexive memos documented coding decisions and disagreement resolution.

| **Domain** | **Code / criterion** | **Definition or analytic use** | **Inclusion rule** | **Exclusion or boundary rule** |
| --- | --- | --- | --- | --- |
| Process code | Protective misrecognition | Symptoms interpreted through safer categories such as chickenpox, allergy, malaria, scabies, or other explanations before mpox is named. | Use when a participant describes alternative naming or delayed acceptance of mpox. | Do not use for confirmed clinical misdiagnosis unless participant frames it as socially safer recognition. |
| Process code | Disclosure risk convergence | The point at which stigma, cost, distance, work risk, relationship risk, and service trust converge around formal presentation or identification. | Use for explicit calculations about what others will know, see, or infer if care is sought. | Do not use for general stigma without connection to care-seeking or identification. |
| Process code | Concealment labour | Active management of illness visibility, stories, clothing, work absence, partners, neighbours, or landlords. | Use when participants describe hiding symptoms or maintaining alternative explanations. | Do not use for private non-disclosure without active management. |
| Process code | Distance as exposure multiplier | Travel, waiting areas, repeated referrals, or visible journeys increase the number of disclosure opportunities. | Use when geographic or transport barriers become socially visible exposure events. | Do not use for distance alone without disclosure visibility. |
| Process code | Health system co-production of delay | Misdiagnosis, unclear referral, delayed tests, limited diagnostic access, or weak confidentiality extend delay after presentation. | Use for system-side contributions to delay or disclosure harm. | Do not use for community-only delay. |
| Process code | Differential diagnosis and red-flag recognition | Need for peripheral health workers to identify possible outbreak conditions and refer safely without expecting perfect diagnosis. | Use for healthcare worker accounts of rash overlap, limited tools, or referral uncertainty. | Do not use to blame junior staff for rare-event diagnosis. |
| Process code | Contact tracing as disclosure work | Tracing changes who knows, when they know, and what they infer; it can protect or amplify stigma. | Use for contact naming, workplace visits, household approaches, confidentiality, or tracing visibility. | Do not use for tracing logistics without disclosure implications. |
| Process code | Trusted intermediary | A person who receives first disclosure and absorbs part of social cost through interpretation, accompaniment, transport, advocacy, or confidentiality negotiation. | Use for nurses, relatives, friends, CHWs, contact tracers, or trusted clinicians serving as disclosure brokers. | Do not use for general advice without relational trust. |
| Process code | Visible confidentiality | Confidentiality made credible through observable facility or field practice. | Use when privacy is demonstrated in triage, waiting, referrals, tracing visits, or staff conduct. | Do not use for generic confidentiality statements. |
| Process code | Actionable communication | Practical messages that connect symptoms to what to do and where to go. | Use when communication enables recognition and same-day or earlier presentation. | Do not use for generic awareness with no care pathway. |
| Typology criterion | Complete trajectory | Account describes illness onset through formal care-seeking or continued non-seeking. | Required for pathway assignment. | Accounts lacking onset-to-care sequence are contextual evidence only. |
| Typology criterion | Disclosure risk detail | Account explicitly refers to social dimensions of disclosure risk. | Required for pathway assignment. | Accounts describing only symptoms or treatment without disclosure risk are contextual evidence only. |
| Typology criterion | Intermediary detail | Account gives enough information on presence or absence of a trusted intermediary. | Required for pathway assignment. | Accounts without intermediary information are contextual evidence only. |
| Pathway type | Supported disclosure | A trusted person absorbs part of the social cost before or during formal care-seeking. | Classify when private disclosure to a trusted person enables supported presentation or safer management. | Do not classify if support occurs only after involuntary exposure. |
| Pathway type | Forced concealment | No trusted disclosure relationship is available; concealment continues until physically or socially impossible. | Classify when presentation or identification is driven by collapse of concealment or external exposure. | Do not classify if early trusted support enabled voluntary care-seeking. |
| Pathway type | Transitional case | Initial concealment shifts toward supported disclosure after a trusted intermediary emerges mid-trajectory. | Classify within forced concealment count if trajectory began with concealment, and analyse separately as pathway modifiability. | Do not merge with supported disclosure if the initial trajectory was already forced concealment. |
| Evidence category | Contextual evidence | Survivor or contextual transcript contributes to process analysis but lacks criteria for pathway assignment. | Use for unclassified survivor accounts and contextual transcripts from HCWs, contact tracers, and community members. | Do not count as pathway cases. |
