# Supplementary material for: Tell people and lose what I have: Care-seeking, concealment, and disclosure during Sierra Leone’s 2025 clade IIb mpox outbreak
Source: PLOS Glob Public Health. 2026 Sep 18;6(9):e0007337. doi: 10.1371/journal.pgph.0007337 (PMC13588523; doi:10.1371/journal.pgph.0007337)
Supplement: Supplementary file 3 [file pgph.0007337.s003.docx]

**Supporting Information**

Tell people and lose what I have: care-seeking, concealment, and disclosure during Sierra Leone’s 2025 clade IIb mpox outbreak

Eric Nzirakaindi Ikoona, Lucy Namulemo, Mary Magdalene Sinnah, Mohamed Alex Vandi, Foday Sahr

**S1 Appendix. Semi-structured interview and focus group discussion guides used to collect survivor, healthcare worker, contact tracer, and community member data for the parent study and this focused analysis**

These guides were used in the parent qualitative study and informed the focused secondary analysis. The appendix contains five guides: interview guides for mpox survivors, healthcare workers, contact tracers, and community members, and the focus group discussion guide used with healthcare workers, community members, and contact tracers. Guides were adapted by stakeholder group and language, with probes used flexibly according to participant responses and sensitivity of the topic.

Participant labels attached to quotations in the manuscript follow a fixed scheme. The data source is a semi-structured interview (SSI) or a focus group discussion (FGD). Role codes are survivor (S), healthcare worker (HCW), contact tracer (CT), and community member (CM). District codes are Western Area Urban (WAU), Western Area Rural (WAR), Bo (BO), and Kenema (KEN). Healthcare worker labels add the cadre at the end.

**Interview guide for mpox survivors**

**Introduction and rapport building**

Thank you for agreeing to speak with me today. I am a trained member of the research team from the National Public Health Agency. We conduct this study to understand the experiences of people who experienced mpox in Sierra Leone. Your participation is voluntary. You can stop at any time or skip any question. We keep everything you share confidential and remove personal identifiers from transcripts. Do you have any questions before we begin?

**Section 1: Illness onset and trajectory**

1. Can you tell me about when you first started feeling unwell? What symptoms did you notice first?

2. What did you think was happening to you at that time?

3. Can you describe how your illness progressed over time?

4. What was the most difficult part of being ill with mpox?

Probes: Physical symptoms, pain, limitations on activities, emotional aspects.

**Section 2: Healthcare-seeking experiences**

5. Can you walk me through what you did when you realised you might need medical care?

6. What factors influenced your decision about when and where to seek care?

7. Can you describe your experiences at the healthcare facility?

8. How did healthcare workers treat you?

Probes: Wait times, privacy, communication, respect, quality of care.

**Section 3: Social responses and stigma**

9. Who did you tell about your diagnosis? How did you decide who to tell?

10. How did your family react when they learned about your illness?

11. How did people in your community respond?

12. Did you experience any negative treatment because of having mpox? If so, can you describe what happened?

Probes: Rejection, avoidance, discrimination, blame, isolation.

**Section 4: Coping and support**

13. What helped you cope during your illness and recovery?

14. Who supported you the most during this time?

15. What additional support would have been helpful?

**Section 5: Recovery and ongoing impacts**

16. How are you feeling now that you have recovered?

17. Are there any lasting effects from your illness, physical, emotional, or social?

18. How has this experience changed you or your life?

**Section 6: Recommendations**

19. Based on your experience, what advice would you give to someone who thinks they might have mpox?

20. What could be done to improve the experience for people with mpox in Sierra Leone?

21. Is there anything else you would like to share about your experience?

**Interview guide for healthcare workers**

**Introduction**

Thank you for agreeing to participate in this study. We are interested in understanding the experiences of healthcare workers involved in the mpox response in Sierra Leone. Your responses will be kept confidential.

**Section 1: Background and role**

1. Can you tell me about your role in the mpox response?

2. How did you become involved in mpox care or response?

3. What training did you receive for working with mpox patients?

**Section 2: Clinical experiences**

4. Can you describe what it has been like caring for mpox patients?

5. What have been the most challenging aspects of providing care?

6. Have there been situations where you felt unable to provide the care you wanted to? If so, can you describe these?

Probes: Resource constraints, knowledge gaps, ethical dilemmas.

**Section 3: Occupational concerns**

7. What concerns, if any, do you have about your own health while working with mpox patients?

8. Do you feel adequately protected? Why or why not?

9. Have you been vaccinated against mpox? Can you tell me about that experience?

**Section 4: Emotional and social impacts**

10. How has working in the mpox response affected you emotionally?

11. How have your family and friends responded to your work in mpox care?

12. Have you experienced any negative treatment from others because of your work?

**Section 5: Observations about patients and community**

13. From your observations, how are mpox patients treated by their families and communities?

14. What barriers do you observe affecting patients’ access to care?

15. How do patients seem to cope with their illness and any stigma they experience?

**Section 6: Recommendations**

16. What additional resources or support would help you in your work?

17. What recommendations do you have for improving the mpox response?

18. Is there anything else you would like to share?

**Interview guide for contact tracers**

**Introduction**

Thank you for agreeing to speak with us. We are interested in the experiences of contact tracers who worked during the mpox outbreak in Sierra Leone. Your participation is voluntary and your responses will be kept confidential. You can stop at any time or skip any question.

**Section 1: Role and training**

1. Can you describe your role in the mpox response and how long you have worked as a contact tracer?

2. What training or preparation did you receive before you began tracing mpox contacts?

3. How did tracing for mpox compare with tracing you had done for other diseases?

Probes: Ebola, COVID-19, caseload, supervision, tools used.

**Section 2: Approaching cases and listing contacts**

4. Can you walk me through what happens from the moment a case is assigned to you?

5. How do you introduce yourself and explain why you are there?

6. How willing are cases to name their contacts?

7. Are there contacts people are more reluctant to name than others? Can you describe those situations?

Probes: sexual partners, household members, employers, neighbours, reasons given.

**Section 3: Disclosure, privacy, and confidentiality**

8. What do people say they are afraid of when you ask them to name contacts?

9. How do you handle a visit when other people are present or watching?

10. What do you do to protect a person’s privacy during a visit?

11. How is information about cases and contacts recorded, stored, and shared?

Probes: workplace visits, doorstep conversations, transport and clothing, telephone contact, forms and registers.

**Section 4: Community response and safety**

12. How do communities react when tracing teams arrive?

13. Have you or your colleagues faced hostility, refusal, or threats? Can you describe what happened?

14. What effect do you think tracing has on how people are treated by their neighbours or employers?

Probes: rumours, avoidance, loss of work, household consequences.

**Section 5: Follow-up and support**

15. What makes follow-up over the monitoring period easy or difficult?

16. What support did you receive for your own wellbeing and safety?

**Section 6: Recommendations**

17. What would make contact tracing safer or more acceptable for the people you visit?

18. Is there anything else you would like to share?

**Interview guide for community members**

**Introduction**

Thank you for agreeing to speak with us. We are interested in how people in your community experienced the mpox outbreak in Sierra Leone. Your participation is voluntary and your responses will be kept confidential. You can stop at any time or skip any question.

**Section 1: Awareness and information**

1. When did you first hear about mpox, and what did you hear?

2. Where did information about mpox come from in your community?

3. What did people believe about how mpox spreads and who gets it?

Probes: radio, social media, health workers, religious and community leaders, rumours.

**Section 2: Observing illness and care-seeking**

4. Did you know anyone in your community who became ill with mpox or was named as a contact?

5. What did you see happen when someone was identified?

6. What did people do when they suspected they had mpox?

Probes: delay, self-treatment, pharmacies, traditional healers, hiding symptoms.

**Section 3: Social response and stigma**

7. How were people with mpox, and their families, treated by neighbours, employers, and landlords?

8. Did anything change for them after they recovered?

9. Were men and women treated differently? Were younger people treated differently from older people?

Probes: avoidance, blame, gossip, assumptions about sexual behaviour, loss of work or housing.

**Section 4: Tracing, isolation, and trust**

10. What did people in the community think about contact tracing and isolation?

11. How did earlier outbreaks affect how people responded this time?

Probes: Ebola, COVID-19, ambulances, home visits, isolation practices.

**Section 5: Support and recommendations**

12. Who helped people who were ill or isolated, and how?

13. What would make it easier for someone in your community to seek care early?

14. Is there anything else you would like to share?

**Focus group discussion guide for healthcare workers, community members, and contact tracers**

**Opening and ground rules**

1. Please introduce yourself, using first name or pseudonym, and your role in the community or response.

2. We discuss experiences of mpox in Sierra Leone. You can decline any question. Please respect confidentiality and do not share other participants’ comments outside this group.

**Core questions**

3. What did people in this community or workplace understand about mpox during the outbreak?

4. What fears or concerns did you see, and what drove them?

5. What forms of stigma or discrimination did you observe in families, communities, workplaces, or facilities?

6. How did stigma affect disclosure, care-seeking, and contact tracing?

7. What helped people seek care or support others, including facilitators, trusted messengers, and services?

8. What barriers to care or response efforts mattered most, including costs, transport, information, privacy, and facility capacity?

9. What strategies reduced stigma or improved care and response coordination?

10. What practical recommendations do you propose for the next outbreak phase?

**Group-specific probes**

Healthcare workers: IPC constraints, workload, moral distress, training, vaccine access, patient communication, privacy.

Community members: rumours and media, social norms, gender and age dynamics, community leadership, support networks.

Contact tracers: disclosure barriers, safety concerns, trust, data privacy, household dynamics, follow-up challenges.

**Closing**

11. What single change would most improve response and reduce stigma?

12. Is there anything we did not ask that matters?
