# Supplementary material for: Tell people and lose what I have: Care-seeking, concealment, and disclosure during Sierra Leone’s 2025 clade IIb mpox outbreak
Source: PLOS Glob Public Health. 2026 Sep 18;6(9):e0007337. doi: 10.1371/journal.pgph.0007337 (PMC13588523; doi:10.1371/journal.pgph.0007337)
Supplement: Supplementary file 5 [file pgph.0007337.s006.docx]

**Response to Reviewers**

Manuscript ID: PGPH-D-26-01372R1

Title: Tell people and lose what I have: care-seeking, concealment, and disclosure during Sierra Leone’s 2025 clade IIb mpox outbreak

We thank the editor and the two reviewers for their comments. Each point is answered below, with the change made and the section where it appears.

**Reviewer #1**

**Comment 1:** Reference 13 (the parent study, Ikoona et al.) currently reads “doi: (to be completed on publication)”. Please replace with the assigned DOI or a preprint identifier before typesetting, so that readers can locate the parent study and verify the “focused secondary analysis” framing.

**Response:** Reference 13 now carries the full published citation and DOI of the parent study: Ikoona EN, Namulemo L, Sinnah MM, Vandi MA, Sahr F. Suffering in silence: stigma, healthcare barriers, and resilience during Sierra Leone’s 2025 clade IIb mpox outbreak—a multi-perspective qualitative study. PLOS Glob Public Health. 2026;6(6):e0006686. doi:10.1371/journal.pgph.0006686. The entry is numbered and formatted in the same style as the rest of the reference list. The Methods describe the parent study as published rather than in production, so a reader can locate it and verify the relationship between the parent study and this focused secondary analysis.

**Comment 2:** The Data Availability Statement in the tracked version refers to the “Ministry of Health and Sanitation” while the clean version refers to the “Ministry of Health”. Please harmonise across both files.

**Response:** The former name Ministry of Health and Sanitation appears nowhere in either file. The Data Availability Statement now directs requests to the Sierra Leone Ethics and Scientific Review Committee through the NPHA institutional contact, without naming the ministry, and reference 8 uses the ministry’s current name, Ministry of Health, Sierra Leone. The clean file, the marked-up file, and the Data Availability field of the submission system carry the same wording.

**Comment 3:** In the Methods section on analytic sufficiency, the statement that coding of “the final 17 survivor transcripts produced no new process codes or pathway features” is a useful indicator of informational sufficiency. A single clarifying sentence stating how the coding order was determined would prevent any appearance of post-hoc saturation reasoning.

**Response:** The Analysis section now states how the coding order was fixed. Survivor transcripts were coded in the chronological order in which the parent study collected them. The codebook and the three pathway criteria were established on the first 25 survivor transcripts, and coding of the remaining 17 produced no new process codes and no new pathway features. The order therefore followed the collection sequence rather than the analytic result, and the two figures sum to the 42 survivor interviews in the focused dataset. The section retains the statement that subgroup numbers were too small to support saturation within gender, age, or district strata.

**Comment 4:** Table 1, “Typical timing” row: please add a one-line footnote stating whether the timing observations are based on survivor recall, contact tracer records, or clinical records.

**Response:** A note now sits directly below Table 1 and states that timing categories are based on survivor-reported illness onset and care-seeking trajectories described during individual interviews. The note also defines n and gives the denominator of 28 of 42 survivor accounts that met all three pathway criteria.

**Comment 5:** In the Discussion section “Transferability and scope conditions”, the citations to tuberculosis, mpox in MSM contexts, and mental health presentations are currently clustered. A brief individual justification for each scope condition would strengthen the transferability argument.

**Response:** The section states the three scope conditions and then justifies each case in turn. Tuberculosis is justified by prolonged diagnostic uncertainty and the social consequences of being identified as infectious. HIV testing and care is justified by the weighing of anticipated social consequences before health information is revealed. Mpox in MSM contexts is justified by visible symptoms combined with sexualised interpretation. Mental health presentations are justified by the risk of social judgement and altered identity within family and community networks. The section closes with the boundary condition that disclosure may not organise care-seeking in the same way where stigma is low and confidentiality is trusted.

**Closing remark:** We are grateful for the assessment that the revision addresses the previous round of review and for the recommendation that the paper be accepted.

**Reviewer #2**

**Comment 1:** Include Sierra Leone locations, Western Area Urban, Western Area Rural, Bo, and Kenema, in the abstract.

**Response:** The abstract names Western Area Urban, Western Area Rural, Bo, and Kenema, together with the data collection period of 10 March to 28 August 2025.

**Comment 2:** Rephrase the statement in the abstract about the focused dataset. What is meant by “contextual transcripts” and by “42 survivor individual interview transcripts”, and the later figures of 28, 16, 12 and 14, which could be summarised.

**Response:** The sentence the reviewer quoted has been rewritten. The abstract now reports the dataset in three short sentences: the focused dataset held 70 transcripts; all 42 survivor interviews formed the primary evidence; and the remaining 28 were contextual transcripts, drawn from interviews and focus group discussions with healthcare workers, contact tracers, and community members. The later figures are tied to the groups they describe. Twenty-eight of the 42 survivor accounts met the pathway criteria, comprising 16 supported disclosure pathways and 12 forced concealment pathways, and the other 14 lacked one or more pathway criteria and contributed contextual survivor evidence. The Methods give the composition of the 28 contextual transcripts by cadre and method, and the same figures appear in the abstract, Methods, Results, Table 1, and Limitations.

**Comment 3:** The Introduction has some initial negating statements followed by a confirmatory scope, for example “The purpose of this paper is not to reject that evidence” and “Our contribution therefore is not the claim that disclosure is new”. Is that necessary?

**Response:** Both sentences have been removed. The Introduction states the contribution directly and frames the study as extending the existing stigma and disclosure literature. We applied the same principle to the rest of the manuscript, because the construction recurred outside the Introduction. The abstract, the Results, the contribution and convergence subsections of the Discussion, and the Conclusions now carry positive formulations. Negative statements remain only where they report an analytic finding, such as the accounts in which disclosure was not the main driver of delay.

**Comment 4:** What is meant by “formal presentation to services is the social threshold”?

**Response:** The Introduction now defines formal presentation to services as the point at which a person must allow others to know, observe, or infer their illness status, and states that stigma, cost, relationship risk, trust, and service barriers converge at that point. The phrase “social threshold” has been replaced by this definition, which governs the Results and the Discussion, so the idea carries one meaning throughout.

**Comment 5:** The Methods treat healthcare worker, contact tracer, and community accounts as contextual, observational, or normative evidence, while the abstract stated that “The other 14 survivor accounts contributed contextual evidence”. Please align.

**Response:** The manuscript now uses two terms and keeps each to one referent. Contextual transcripts refers to the 28 healthcare worker, contact tracer, and community member sources. Contextual survivor evidence refers to the 14 survivor accounts that did not meet the pathway criteria. The abstract, Methods, Results, and Limitations use the two terms consistently.

**Journal questions**

**Peer review history:** We have completed the submission preference for publication of the peer review history in the journal system.

**Reference list:** We have checked the reference list. Reference 13 now carries the published citation and DOI of the parent study, as described under Reviewer #1, Comment 1. No references were added or removed, and none of the cited works has been retracted.

**Files:** We enclose the clean manuscript, a marked-up copy in which the changed passages are highlighted, and this response letter.

We thank the reviewers for these comments, which have improved the clarity of the numbers, the transparency of the analytic claims, and the precision of the terminology.
