# Supplementary material for: Tell people and lose what I have: Care-seeking, concealment, and disclosure during Sierra Leone’s 2025 clade IIb mpox outbreak
Source: PLOS Glob Public Health. 2026 Sep 18;6(9):e0007337. doi: 10.1371/journal.pgph.0007337 (PMC13588523; doi:10.1371/journal.pgph.0007337)
Supplement: Supplementary file 6 [file pgph.0007337.s005.docx]

# Response to Reviewers

Manuscript ID: PGPH-D-26-01372

Title: Tell people and lose what I have: care-seeking, concealment, and disclosure during Sierra Leone’s 2025 clade IIb mpox outbreak

Dear Dr. Grant and Reviewers,

We thank the editor and reviewers for their careful assessment and constructive recommendations. We revised the manuscript to clarify its relationship to the accepted parent study, deepen the methods, make the focus group and multi-method approach explicit, strengthen qualitative rigour, tone down novelty claims, foreground gender, contact tracing, Ebola and COVID-19 memory, and clarify the supported disclosure versus forced concealment typology. We also updated the data availability statement, financial disclosure, competing interests, supporting information labels, parent-study citation, and PLOS formatting.

# Journal Requirements

**Comment JR1. Please amend the online Financial Disclosure statement.**

Response: We amended the manuscript and will amend the online form to state: “The authors received no specific funding for this work.”

**Comment JR2. Please update the online Competing Interests statement.**

Response: We amended the manuscript and will amend the online form to state: “The authors have declared that no competing interests exist.”

**Comment JR3. Please clarify the Data Availability Statement for qualitative data.**

Response: We revised the Data Availability Statement. Full transcripts cannot be shared publicly because they contain sensitive information and indirect identifiers related to a stigmatising outbreak, geographic location, occupation, household relationships, and contact tracing experiences. We state that de-identified excerpts or a coded framework matrix may be requested under a data-sharing agreement, subject to approval by the Sierra Leone Ethics and Scientific Review Committee, with an institutional contact provided.

**Comment JR4. Ethics approval documents should not be published with the paper and should be uploaded as “Other”.**

Response: We will upload any ethics approval documentation as “Other” so that it is available for editorial review but not published as supporting information.

**Comment JR5. Review any recommended references and cite if relevant.**

Response: We reviewed the recommended disclosure literature and incorporated relevant references, including Chaudoir and Fisher, Obermeyer et al., and related HIV stigma and disclosure literature.

# Reviewer #1

**Comment 1. The novelty claim needs toning down. The HIV disclosure literature has covered this ground for two decades, and the manuscript should explain what the present framework adds beyond applying known concepts to mpox.**

Response: We agree. We revised the Introduction and Discussion to state clearly that disclosure is not a new concept and that the manuscript does not claim novelty for disclosure itself. The contribution is now framed as an outbreak-specific account of how disclosure operates as a convergence point during an acute, visible, sexually moralised, and surveillance-linked outbreak. We added relevant HIV disclosure literature and clarified that the disclosure model extends, rather than replaces, existing stigma and disclosure frameworks.

**Comment 2. Only 28 of 42 survivors could be classified into the typology. What characterised the other 14?**

Response: We added explicit pathway assignment criteria in the Methods and explained the 14 unclassified survivor accounts in the Results. The 14 accounts were not inconsistent with the typology; they lacked one or more criteria required for pathway assignment. Some focused on symptoms and eventual treatment without enough detail on the social decision process; others focused on post-diagnosis stigma, transport or cost barriers, or recovery rather than the complete onset-to-care trajectory. They were therefore used as contextual evidence but not counted as pathway cases.

**Comment 3. Gender is absent from the analysis despite male and female participants and genital involvement being a key feature of clade IIb.**

Response: We expanded the Results and Discussion to include gendered and relational analysis, with illustrative survivor quotations. We now explain that disclosure risks were gendered but not reducible to a simple male-female split. Male survivors more often discussed accusations of sexual misconduct, partnership breakdown, marriage loss, children, standing, and income. Female survivors more often described shame, moral judgement, family rejection, and fear that genital symptoms would be interpreted as unacceptable sexual behaviour. We also discuss intimate partner risk when only one partner showed symptoms.

**Comment 4. The barrier model is set up as a straw man. Modern outbreak frameworks already recognise that barriers interact.**

Response: We revised the framing. The manuscript now states that existing outbreak and care-seeking frameworks recognise interacting barriers and that our contribution is not to reject this literature. Instead, we identify the mechanism and point of convergence: the moment when private illness risks becoming public identity through formal care-seeking, contact tracing, or facility presentation.

**Comment 5. The three transitional cases are arguably the most important finding for intervention design and need more space.**

Response: We expanded the transitional cases in the Results, Table 1, Discussion, and implications. We now explain that transitional cases reveal the key intervention window: people already concealing symptoms may still shift toward care when a trusted intermediary emerges. We emphasise that response systems should create low-exposure routes through which symptomatic people can reach a trusted first contact before concealment becomes involuntary exposure.

**Comment 6. Quotes read very polished for Krio-to-English translation. Disclose degree of editing and back-translation concordance.**

Response: We added a dedicated Methods statement on language, transcription, translation, and quotation presentation. We state that sessions were conducted in Krio, Mende, Temne, or English, transcribed verbatim in the original language, translated by bilingual team members, and back-translated for a random 20% of segments. Back-translation identified minor wording differences but no theme-altering discrepancies. We also state that quotations were lightly edited for readability and removal of identifying details without altering substantive meaning.

**Comment 7. Member checking is mentioned but not described. How many survivors, what feedback?**

Response: We expanded the rigour section. We now state that five survivor participants reviewed summaries of the supported disclosure, forced concealment, and transitional pathways. They confirmed that the typology reflected their experiences and emphasised confidentiality and trusted accompaniment, which were foregrounded in the pathway definitions and implications.

**Comment 8. Table 1 needs case counts: 16 supported, 12 forced, 3 transitional.**

Response: We revised Table 1 to include case counts. The table now states n=16 for supported disclosure, n=12 for forced concealment, and n=3 transitional cases included within the forced concealment count because each began with concealment before shifting.

**Comment 9. The reference list is thin for a conceptual paper.**

Response: We expanded the reference list from 22 to 35 references. We added key disclosure, stigma, qualitative rigour, Ebola memory, contact tracing ethics, healthcare worker mpox, tuberculosis delay, and mpox illness experience literature.

**Comment 10. Abstract likely exceeds the 300-word PLOS limit.**

Response: We revised and shortened the abstract. The current abstract is approximately 269 words and remains within the PLOS 300-word limit.

**Comment 11. Clarify whether de-identified transcripts or the coded framework matrix can be shared under a data-sharing agreement.**

Response: We revised the Data Availability Statement. It now states that full transcripts cannot be shared publicly for confidentiality and re-identification reasons, but additional de-identified excerpts or a coded framework matrix may be requested under a data-sharing agreement, subject to Sierra Leone Ethics and Scientific Review Committee approval.

**Comment 12. Were there survivors for whom disclosure was not the main driver of delay?**

Response: We added this explicitly in the Results. Some survivors emphasised transport cost, physical weakness, the need to maintain daily income, or confusion caused by inconsistent early diagnoses. We also explain that even where disclosure was not the main driver, the social meaning of being seen with mpox often intensified practical barriers.

**Comment 13. How was the PI’s role as Country Technical Director managed regarding participant responses?**

Response: We expanded the positionality and reflexivity section. We now describe the investigator team’s public health roles and the steps used to reduce response and interpretation bias: most data collection was conducted by trained research assistants outside the national response coordination structure; participants were told participation was voluntary and would not affect care, services, surveillance follow-up, or employment; LN provided an external analytic counterweight; and reflexive memos documented and challenged insider assumptions.

**Comment 14. Did healthcare worker accounts ever contradict survivor narratives on reasons for delay?**

Response: We added this in the Results. Healthcare worker accounts did not directly contradict survivor accounts. They described the same encounters from the system side and differed mainly in emphasis, particularly by highlighting diagnostic uncertainty, peripheral facility readiness, and referral limitations.

# Reviewer #2

**Comment 1. Provide detailed information on the data collection process in this sub-study. Clarify how the data instruments were developed and cite supplementary files.**

Response: We expanded the Methods to describe the relationship to the parent study, the focused dataset, source selection, instruments, language, transcription, translation, quotation presentation, analysis, rigour, positionality, and ethics. We also cite S3 Appendix for the data collection instruments.

**Comment 2. Provide information on focus group discussions: size, mix, planning, and execution.**

Response: We added a focus group methods paragraph. We now state that FGDs contained 6–10 participants, lasted approximately 60–90 minutes, were grouped by stakeholder type and district, and were structured to support peer discussion while protecting confidentiality. We explain that no survivor FGDs were held because survivor illness histories, genital symptoms, suspected sexual exposure, and disclosure risks were too sensitive for group discussion. We also describe facilitation practices used to invite quieter participants, encourage disagreement, avoid identifiable disclosures, and offer follow-up individual interviews where needed.

**Comment 3. Explain the reasons for the multi-method data collection approach and how each method contributed to analysis, including handling of FGDs and participant groups.**

Response: We revised the Methods to distinguish epistemic positions. Survivor interviews are treated as first-person care-seeking narratives. Healthcare worker, contact tracer, and community accounts are treated as contextual, observational, or normative evidence. Individual interviews carried the experiential analysis, while FGDs informed collective meaning, operational practice, and points of agreement or disagreement across roles. We compared sources in shared matrices rather than treating FGDs as simple confirmation of interviews.

**Comment 4. Clarify use of memos and saturation, including whether saturation was needed in the main paper or subgroup analyses.**

Response: We added the use of reflexive memos to document analytic decisions, disagreement resolution, the PI’s insider position, and emerging interpretations. Because this was a focused secondary analysis of an existing corpus, we did not claim de novo thematic saturation. We instead assessed analytic sufficiency or information power for the focused question. We also state that subgroup numbers were too small to claim saturation within gender, age, or district strata.

**Comment 5. Qualitative rigour: transferability was not addressed.**

Response: We added a transferability section. The manuscript now addresses transferability through thick description of context, participant groups, outbreak setting, pathway criteria, and explicit scope conditions. The framework is presented as most applicable where diagnosis carries high social stakes, symptoms or care-seeking are visible, and confidentiality systems are weak, mistrusted, or socially observable.

**Comment 6. Was there community engagement or public group participation in research design and instrument development?**

Response: We clarified this in the instrument section. No formal community advisory board co-designed the study. However, the guides were refined with input from Community Health Officers, District Health Management Team staff, treatment centre staff, and pilot participants. We state this clearly to avoid overstating community co-design.

**Comment 7. Indicate which quotations were from interviews and which were from FGDs.**

Response: We revised all quotation labels to indicate SSI or FGD, together with participant role, gender, age where available, and district.

**Comment 8. Reframe expectations around rare outbreak diagnosis, especially among junior health workers.**

Response: We revised the Results and Discussion. We no longer imply that junior health workers should recognise rare events perfectly at early stages. We state that the appropriate expectation is differential diagnosis, red-flag recognition, safe referral, escalation, and diagnostic support when symptoms do not fit routine categories.

**Comment 9. Contextualise sexually transmitted infection and supported disclosure where only one partner shows symptoms and infidelity consequences may be worse.**

Response: We added gendered and intimate partnership analysis. The revised manuscript explains that where one partner showed symptoms and the other did not, mpox could be interpreted as evidence of infidelity or socially unacceptable sexual behaviour, even when the actual transmission route was unknown. We explain how trusted intermediaries helped manage the relational risk of naming mpox, contacting partners, and entering care.

**Comment 10. Elaborate on the role and contribution of contact tracers in disclosure and associated stigma, grounded in Ebola and COVID-19 context.**

Response: We expanded Process 3 and the Discussion. Contact tracing is now analysed as disclosure work. Contact tracers could protect disclosure when they approached people discreetly, explained confidentiality, and acted as trusted intermediaries. They could also amplify stigma when visits to homes or workplaces made exposure visible. We explicitly ground this in Sierra Leone’s Ebola and COVID-19 memories of public health visits, visible isolation, and surveillance signalling illness.

**Comment 11. Ebola and COVID-19 should be central to the discussion of disclosure and non-disclosure.**

Response: We added a dedicated Discussion section titled “Epidemic memory, contact tracing, and non-disclosure.” It explains how Ebola and COVID-19 shaped expectations about public health teams, isolation, ambulance movement, and household identification, which made non-disclosure a rational protective strategy for some participants during the mpox outbreak.

**Comment 12. Explain how previous outbreaks perpetuated non-disclosure in Sierra Leone or the sub-region.**

Response: We now explain that previous outbreaks created social memories of being marked by association, visible surveillance, and public health action that could identify households or individuals before any diagnosis was openly discussed. These memories interacted with sexualised mpox stigma and contributed to concealment and delayed presentation.

# Closing

We appreciate the reviewers’ guidance. The revision is substantially strengthened by their comments, especially in relation to methods transparency, the role of contact tracing, gendered disclosure risk, epidemic memory, and the intervention relevance of transitional cases.
