# Supplementary material for: Tell people and lose what I have: Care-seeking, concealment, and disclosure during Sierra Leone’s 2025 clade IIb mpox outbreak
Source: PLOS Glob Public Health. 2026 Sep 18;6(9):e0007337. doi: 10.1371/journal.pgph.0007337 (PMC13588523; doi:10.1371/journal.pgph.0007337)
Supplement: Peer review history

# Peer review: Decision Letter 0

**DOI:** 10.1371/journal.pgph.0007337.r001

**Authors:** Catherine Grant

17 Jun 2026

PGPH-D-26-01372

Tell people and lose what I have: care-seeking, concealment, and disclosure during Sierra Leone’s 2025 clade IIb mpox outbreak

PLOS Global Public Health

Dear Dr. Ikoona,

Thank you for submitting your manuscript to PLOS Global Public Health. After careful consideration, we feel that it has merit but does not fully meet PLOS Global Public Health’s publication criteria as it currently stands. Therefore, we invite you to submit a revised version of the manuscript that addresses the points raised during the review process.

==============================

The revisions needed, and outlined by the reviewers include:

Methods:

• While the main study is still under review, it is also important to provide detailed information on the data collection process in this sub-study. Clarify how the data instrument for semi-structured interviews and focus group discussions were developed and cite as supplementary files.

• No information on the focus group discussion is provided. How many participants were in each group, and what informed the numbers and mix of participants, how the discussion was planned and executed to get feedback from all participants etc.

• What were the reasons for a multi-method data collection approach, and how did each method contribute to the analysis? Or one served to triangulate the other. unclear how data from different participant groups were handled and how the FGD was analysed. The use of memos and in the analysis. Was there a need to achieve saturation in the main paper or in the subgroup analyses?

• Qualitative Rigour: transferability was not addressed. How could this be assessed/achieved

• Was there any community engagement/involvement or public group participation in developing the research design and instrument?

Results/Discussion:

• It is unclear which quotes were from interviews and from FGD…. it's good to indicate

• Outbreaks are rare events in themselves. The expectation that health workers (particularly junior) may be conversant and updated in diagnosing rare events, especially at the early stages, is overstretched. The framing should sit with health workers able to recognise differential diagnosis and possible referral, especially when diagnostic tools may be absent altogether. This is not limited to a particular setting but has been documented globally in the early stages of a new outbreak in new settings.

• In supported disclosure, it is also important to contextualise sexually transmitted infection (outbreak or not) and only one partner has shown signs of the disease presentation. The repercussions of potential infidelity could be even worse.... any issues came up in the results?

• It is very important to elaborate on the role/contribution of contact tracers in disclosure and associated stigma contributors. This should also be grounded in the context of previous outbreaks, mainly Ebola and COVID-19 in Sierra Leone.

• Ebola and COVID-19 should be a centrepiece in the discussion of disclosure, and those two recent outbreaks set a strong determinant of non-disclosure during outbreaks.

• How has the previous outbreak perpetuated non-disclosure in Sierra Leone/sub-region.

1. The novelty claim needs toning down. The HIV disclosure literature (Chaudoir and Fisher 2010, Obermeyer et al. 2011) has covered this ground for two decades. The paper cites some of it but does not explain what the present framework actually adds beyond applying known concepts to mpox.

2. Only 28 of 42 survivors could be classified into the typology. What characterised the other 14? This is a third of the sample unclassified, and the authors propose this as a planning tool.

3. Gender is absent from the analysis despite having male and female participants and genital involvement being a key feature of clade IIb. Disclosure risks are obviously gendered.

4. The barrier model is set up as a straw man. Modern outbreak frameworks already recognise that barriers interact. The argument is stronger when identifying the mechanism of convergence rather than pretending the field treats barriers as independent.

5. The three transitional cases (concealment shifting to supported disclosure) are mentioned in one sentence but are arguably the most important finding for intervention design. Give them more space.

6. Quotes read very polished for Krio-to-English translation. Disclose degree of editing and back-translation concordance.

7. Member checking is mentioned but not described. How many survivors, what feedback?

8. Table 1 needs case counts (16 supported, 12 forced, 3 transitional).

9. Reference list is thin for a conceptual paper (22 references).

10. Abstract likely exceeds the 300-word PLOS limit.

Questions for authors

11. The Data Availability Statement says all relevant data are included within the manuscript and additional materials can be provided upon reasonable request. For qualitative research with stigmatised populations this is acceptable, but the authors should clarify whether de-identified transcripts or the coded framework matrix can be shared under a data-sharing agreement, since the codebook is provided as supplementary material but the underlying data are not.

12. Were there survivors for whom disclosure was not the main driver of delay?

13. How was the PI's role as Country Technical Director managed regarding participant responses?

14. Did healthcare worker accounts ever contradict survivor narratives on reasons for delay?

==============================

Please submit your revised manuscript by Aug 16 2026 11:59PM. If you will need more time than this to complete your revisions, please reply to this message or contact the journal office at globalpubhealth@plos.org. When you're ready to submit your revision, log on to https://www.editorialmanager.com/pgph/ and select the 'Submissions Needing Revision' folder to locate your manuscript file.

Please include the following items when submitting your revised manuscript:

- A letter that responds to each point raised by the editor and reviewer(s). You should upload this letter as a separate file labeled 'Response to Reviewers'.
- A marked-up copy of your manuscript that highlights changes made to the original version. You should upload this as a separate file labeled 'Revised Manuscript with Track Changes'.
- An unmarked version of your revised paper without tracked changes. You should upload this as a separate file labeled 'Manuscript'.

Guidelines for resubmitting your figure files are available below the reviewer comments at the end of this letter.

As the corresponding author, your ORCID iD is verified in the submission system and will appear in the published article. PLOS supports the use of ORCID, and we encourage all coauthors to register for an ORCID iD and use it as well. Please encourage your coauthors to verify their ORCID iD within the submission system before final acceptance, as unverified ORCID iDs will not appear in the published article. *Only*  the individual author can complete the verification step; PLOS staff *cannot*  verify ORCID iDs on behalf of authors.

We look forward to receiving your revised manuscript.

Kind regards,

Catherine Grant

Academic Editor

PLOS Global Public Health

Journal Requirements:

1. Please amend your online Financial Disclosure statement. If you did not receive any funding for this study, please simply state: "The authors received no specific funding for this work."

2. Please update your online Competing Interests statement. If you have no competing interests to declare, please state: "The authors have declared that no competing interests exist."

3. In the online submission form, you indicated that "All relevant data supporting the findings of this study are included within the manuscript. Any additional materials can be made available by the corresponding author upon reasonable request.".

All PLOS journals now require all data underlying the findings described in their manuscript to be freely available to other researchers, either

a) In a public repository,

b) Within the manuscript itself, or

c) Uploaded as supplementary information.

This policy applies to all data except where public deposition would breach compliance with the protocol approved by your research ethics board. If your data cannot be made publicly available for ethical or legal reasons (e.g., public availability would compromise patient privacy), please explain your reasons by return email and your exemption request will be escalated to the editor for approval. Your exemption request will be handled independently and will not hold up the peer review process, but will need to be resolved should your manuscript be accepted for publication. One of the Editorial team will then be in touch if there are any issues.

For further assistance, you may go to: http://journals.plos.org/globalpublichealth/s/data-availability

4. We note that the ethics approval documents should not be published with the paper and are for internal information only. Please change the file type to 'Other' to ensure that they are not published.

5. If the reviewer comments include a recommendation to cite specific previously published works, please review and evaluate these publications to determine whether they are relevant and should be cited. There is no requirement to cite these works unless the editor has indicated otherwise.

Additional Editor Comments (if provided):

[Note: HTML markup is below. Please do not edit.]

Reviewers' comments:

Reviewer's Responses to Questions

**Comments to the Author**

1. Does this manuscript meet PLOS Global Public Health’s publication criteria?

Reviewer #1: Yes

Reviewer #2: Yes

**********

2. Has the statistical analysis been performed appropriately and rigorously?

Reviewer #1: N/A

Reviewer #2: N/A

**********

3. Have the authors made all data underlying the findings in their manuscript fully available (please refer to the Data Availability Statement at the start of the manuscript PDF file)?

The PLOS Data policy

Reviewer #1: No

Reviewer #2: No

**********

4. Is the manuscript presented in an intelligible fashion and written in standard English?

Reviewer #1: Yes

Reviewer #2: Yes

**********

Reviewer #1: Good qualitative study, useful reframing of care-seeking as a disclosure event, strong participant voices, large sample for qualitative work.

Major comments

1. The novelty claim needs toning down. The HIV disclosure literature (Chaudoir and Fisher 2010, Obermeyer et al. 2011) has covered this ground for two decades. The paper cites some of it but does not explain what the present framework actually adds beyond applying known concepts to mpox.

2. Only 28 of 42 survivors could be classified into the typology. What characterised the other 14? This is a third of the sample unclassified, and the authors propose this as a planning tool.

3. Gender is absent from the analysis despite having male and female participants and genital involvement being a key feature of clade IIb. Disclosure risks are obviously gendered.

4. The barrier model is set up as a straw man. Modern outbreak frameworks already recognise that barriers interact. The argument is stronger when identifying the mechanism of convergence rather than pretending the field treats barriers as independent.

5. The three transitional cases (concealment shifting to supported disclosure) are mentioned in one sentence but are arguably the most important finding for intervention design. Give them more space.

Minor comments

6. Quotes read very polished for Krio-to-English translation. Disclose degree of editing and back-translation concordance.

7. Member checking is mentioned but not described. How many survivors, what feedback?

8. Table 1 needs case counts (16 supported, 12 forced, 3 transitional).

9. Reference list is thin for a conceptual paper (22 references).

10. Abstract likely exceeds the 300-word PLOS limit.

Questions for authors

11. The Data Availability Statement says all relevant data are included within the manuscript and additional materials can be provided upon reasonable request. For qualitative research with stigmatised populations this is acceptable, but the authors should clarify whether de-identified transcripts or the coded framework matrix can be shared under a data-sharing agreement, since the codebook is provided as supplementary material but the underlying data are not.

12. Were there survivors for whom disclosure was not the main driver of delay?

13. How was the PI's role as Country Technical Director managed regarding participant responses?

14. Did healthcare worker accounts ever contradict survivor narratives on reasons for delay?

Reviewer #2: General:

The paper is important in how we frame factors influencing care-seeking behaviours during outbreaks across multiple settings, but also routine care for stigma-associated conditions or presentations. While the barrier model is important and explored extensively, I do appreciate the author's novelty in exploring a new approach – disclosure, or I would say an intrinsic determinant of the barrier model itself, but as an important addition to the literature.

Several issues need to be considered to improve this piece of communication. Below, I raise them without particular order

Methods:

• While the main study is still under review, it is also important to provide detailed information on the data collection process in this sub-study. Clarify how the data instrument for semi-structured interviews and focus group discussions were developed and cite as supplementary files.

• No information on the focus group discussion is provided. How many participants were in each group, and what informed the numbers and mix of participants, how the discussion was planned and executed to get feedback from all participants etc.

• What were the reasons for a multi-method data collection approach, and how did each method contribute to the analysis? Or one served to triangulate the other. unclear how data from different participant groups were handled and how the FGD was analysed. The use of memos and in the analysis. Was there a need to achieve saturation in the main paper or in the subgroup analyses?

• Qualitative Rigour: transferability was not addressed. How could this be assessed/achieved

• Was there any community engagement/involvement or public group participation in developing the research design and instrument?

Results/Discussion:

• It is unclear which quotes were from interviews and from FGD…. it's good to indicate

• Outbreaks are rare events in themselves. The expectation that health workers (particularly junior) may be conversant and updated in diagnosing rare events, especially at the early stages, is overstretched. The framing should sit with health workers able to recognise differential diagnosis and possible referral, especially when diagnostic tools may be absent altogether. This is not limited to a particular setting but has been documented globally in the early stages of a new outbreak in new settings.

• In supported disclosure, it is also important to contextualise sexually transmitted infection (outbreak or not) and only one partner has shown signs of the disease presentation. The repercussions of potential infidelity could be even worse.... any issues came up in the results?

• It is very important to elaborate on the role/contribution of contact tracers in disclosure and associated stigma contributors. This should also be grounded in the context of previous outbreaks, mainly Ebola and COVID-19 in Sierra Leone.

• Ebola and COVID-19 should be a centrepiece in the discussion of disclosure, and those two recent outbreaks set a strong determinant of non-disclosure during outbreaks.

• How has the previous outbreak perpetuated non-disclosure in Sierra Leone/sub-region.

**********

what does this mean?). If published, this will include your full peer review and any attached files.

**Do you want your identity to be public for this peer review?**  If you choose “no”, your identity will remain anonymous but your review may still be made public.

For information about this choice, including consent withdrawal, please see our Privacy Policy

Reviewer #1: No

Reviewer #2: **Yes:**  Mamadu Baldeh

**********

[NOTE: If reviewer comments were submitted as an attachment file, they will be attached to this email and accessible via the submission site. Please log into your account, locate the manuscript record, and check for the action link "View Attachments". If this link does not appear, there are no attachment files.]

# Author comment: Author response to Decision Letter 1

**DOI:** 10.1371/journal.pgph.0007337.r002

19 Jun 2026

Attachment Submitted filename: PGPH-D-26-01372_Response_to_Reviewers.docx

# Peer review: Decision Letter 1

**DOI:** 10.1371/journal.pgph.0007337.r003

**Authors:** Steve Zimmerman

25 Aug 2026

PGPH-D-26-01372R1

Tell people and lose what I have: care-seeking, concealment, and disclosure during Sierra Leone’s 2025 clade IIb mpox outbreak

PLOS Global Public Health

Dear Dr. Ikoona,

Thank you for submitting your manuscript to PLOS Global Public Health. After careful consideration, we feel that it has merit but does not fully meet PLOS Global Public Health’s publication criteria as it currently stands. Therefore, we invite you to submit a revised version of the manuscript that addresses the points raised during the review process.

The manuscript has been evaluated by two reviewers, and their comments are available below.

Could you please revise the manuscript to carefully address the concerns raised?

Please submit your revised manuscript by Sep 24 2026 11:59PM. If you will need more time than this to complete your revisions, please reply to this message or contact the journal office at globalpubhealth@plos.org. When you're ready to submit your revision, log on to https://www.editorialmanager.com/pgph/ and select the 'Submissions Needing Revision' folder to locate your manuscript file.

Please include the following items when submitting your revised manuscript:

- A letter that responds to each point raised by the editor and reviewer(s). You should upload this letter as a separate file labeled 'Response to Reviewers'.
- A marked-up copy of your manuscript that highlights changes made to the original version. You should upload this as a separate file labeled 'Revised Manuscript with Track Changes'.
- An unmarked version of your revised paper without tracked changes. You should upload this as a separate file labeled 'Manuscript'.

Guidelines for resubmitting your figure files are available below the reviewer comments at the end of this letter.

As the corresponding author, your ORCID iD is verified in the submission system and will appear in the published article. PLOS supports the use of ORCID, and we encourage all coauthors to register for an ORCID iD and use it as well. Please encourage your coauthors to verify their ORCID iD within the submission system before final acceptance, as unverified ORCID iDs will not appear in the published article. *Only*  the individual author can complete the verification step; PLOS staff *cannot*  verify ORCID iDs on behalf of authors.

We look forward to receiving your revised manuscript.

Kind regards,

Steve Zimmerman, PhD

PLOS Staff Editor

**Journal Requirements:**

If the reviewer comments include a recommendation to cite specific previously published works, please review and evaluate these publications to determine whether they are relevant and should be cited. There is no requirement to cite these works unless the editor has indicated otherwise.

Please review your reference list to ensure that it is complete and correct. If you have cited papers that have been retracted, please include the rationale for doing so in the manuscript text, or remove these references and replace them with relevant current references. Any changes to the reference list should be mentioned in the rebuttal letter that accompanies your revised manuscript. If you need to cite a retracted article, indicate the article’s retracted status in the References list and also include a citation and full reference for the retraction notice.

**Additional Editor Comments (if provided):**

[Note: HTML markup is below. Please do not edit.]

Reviewers' comments:

Reviewer's Responses to Questions

**Comments to the Author**

Reviewer #1: All comments have been addressed

Reviewer #2: All comments have been addressed

**********

publication criteria?

Reviewer #1: Yes

Reviewer #2: Yes

**********

3. Has the statistical analysis been performed appropriately and rigorously?

Reviewer #1: N/A

Reviewer #2: N/A

**********

4. Have the authors made all data underlying the findings in their manuscript fully available (please refer to the Data Availability Statement at the start of the manuscript PDF file)?

The PLOS Data policy

Reviewer #1: Yes

Reviewer #2: Yes

**********

5. Is the manuscript presented in an intelligible fashion and written in standard English?

Reviewer #1: Yes

Reviewer #2: Yes

**********

**Reviewer #1:**  The authors have engaged thoroughly with the previous round of review. The revision is substantially strengthened: the novelty claim is appropriately toned down, disclosure is positioned as a mid-range framework that extends rather than replaces the HIV disclosure and stigma literature, and the relationship to the accepted parent study is now transparent. The gendered analysis, the treatment of contact tracing as disclosure work, the framing of Ebola and COVID-19 as epidemic memory, and the expanded analytic account of the three transitional cases all address my previous concerns.

A small number of minor points remain, all of which can be addressed at proof stage without further peer review:

- Reference 13 (the parent study, Ikoona et al.) currently reads "doi: (to be completed on publication)". Please replace with the assigned DOI or a preprint identifier before typesetting, so that readers can locate the parent study and verify the "focused secondary analysis" framing.

- The Data Availability Statement in the tracked version refers to the "Ministry of Health and Sanitation" while the clean version refers to the "Ministry of Health". Please harmonise across both files.

- In the Methods section on analytic sufficiency, the statement that coding of "the final 17 survivor transcripts produced no new process codes or pathway features" is a useful indicator of informational sufficiency. A single clarifying sentence stating how the coding order was determined would prevent any appearance of post-hoc saturation reasoning.

- Table 1, "Typical timing" row: please add a one-line footnote stating whether the timing observations are based on survivor recall, contact tracer records, or clinical records.

- In the Discussion section "Transferability and scope conditions", the citations to tuberculosis, mpox in MSM contexts, and mental health presentations are currently clustered. A brief individual justification for each scope condition would strengthen the transferability argument.

- The paper makes a clear, situated contribution. It does not overclaim disclosure as novel and it demonstrates convincingly how the disclosure threshold organises care-seeking during an acute, visible, sexually moralised outbreak with strong epidemic memory. The supported disclosure versus forced concealment typology, with the transitional cases as an intervention window, is analytically useful and operationally actionable for outbreak preparedness in high-stigma settings.

The paper is a valuable contribution to PLOS Global Public Health and should be accepted.

**Reviewer #2:**  Thank you for the substantial revisions. The manuscript has improved considerably.

Minor comments

1. Include Sierra Leone...'Western Area Urban, Western Area Rural, Bo, and Kenema".... in the abstract.

2. Rephrase the statement (Abstract): 'The focused dataset included all 42 survivor individual interview transcripts and 28 contextual transcripts from healthcare workers, contact tracers, and community members, including individual interviews and focus group discussions'. what is 'contextual transcripts', '42 survivor individual interview transcripts' later: 28, 16, 12 and 14... These could be summarised.

3. The introduction have some initial 'negating pretext' statements followed by a 'confirmatory' scope... is that necessary: 'The purpose of this paper is not to reject that evidence.', 'Our contribution therefore is not the

50 claim that disclosure is new.'

4. 'formal presentation to services is the social threshold', meaning?

5. "We treated survivor interviews as first-person care-seeking narratives and treated healthcare worker, contact tracer, and community accounts as contextual, observational, or normative evidence." but the abstract stated "The other 14 survivor accounts contributed contextual evidence." please allign

**********

what does this mean?). If published, this will include your full peer review and any attached files.

**Do you want your identity to be public for this peer review?**  If you choose “no”, your identity will remain anonymous but your review may still be made public.

For information about this choice, including consent withdrawal, please see our Privacy Policy

Reviewer #1: No

Reviewer #2: No

**********

[NOTE: If reviewer comments were submitted as an attachment file, they will be attached to this email and accessible via the submission site. Please log into your account, locate the manuscript record, and check for the action link "View Attachments". If this link does not appear, there are no attachment files.]

**Figure Resubmissions:**

# Author comment: Author response to Decision Letter 2

**DOI:** 10.1371/journal.pgph.0007337.r004

26 Aug 2026

Attachment Submitted filename: Response_to_Reviewers.docx

# Peer review: Decision Letter 2

**DOI:** 10.1371/journal.pgph.0007337.r005

**Authors:** Maria Carinnes Alejandria

2 Sep 2026

Tell people and lose what I have: care-seeking, concealment, and disclosure during Sierra Leone’s 2025 clade IIb mpox outbreak

PGPH-D-26-01372R2

Dear Dr Ikoona,

We are pleased to inform you that your manuscript 'Tell people and lose what I have: care-seeking, concealment, and disclosure during Sierra Leone’s 2025 clade IIb mpox outbreak' has been provisionally accepted for publication in PLOS Global Public Health.

Before your manuscript can be formally accepted you will need to complete some formatting changes, which you will receive in a follow up email. A member of our team will be in touch with a set of requests.

Please note that your manuscript will not be scheduled for publication until you have made the required changes, so a swift response is appreciated.

IMPORTANT: The editorial review process is now complete. PLOS will only permit corrections to spelling, formatting or significant scientific errors from this point onwards. Requests for major changes, or any which affect the scientific understanding of your work, will cause delays to the publication date of your manuscript.

If your institution or institutions have a press office, please notify them about your upcoming paper to help maximize its impact. If they'll be preparing press materials, please inform our press team as soon as possible -- no later than 48 hours after receiving the formal acceptance. Your manuscript will remain under strict press embargo until 2 pm Eastern Time on the date of publication. For more information, please contact globalpubhealth@plos.org.

Thank you again for supporting Open Access publishing; we are looking forward to publishing your work in PLOS Global Public Health.

Best regards,

Maria Carinnes Alejandria

Academic Editor

PLOS Global Public Health

***********************************************************

Reviewer Comments (if any, and for reference):

Reviewer's Responses to Questions

**Comments to the Author**

Reviewer #1: All comments have been addressed

**********

publication criteria?

Reviewer #1: Yes

**********

3. Has the statistical analysis been performed appropriately and rigorously?

Reviewer #1: N/A

**********

4. Have the authors made all data underlying the findings in their manuscript fully available (please refer to the Data Availability Statement at the start of the manuscript PDF file)?

The PLOS Data policy

Reviewer #1: Yes

**********

5. Is the manuscript presented in an intelligible fashion and written in standard English?

Reviewer #1: Yes

**********

Reviewer #1: All five points I raised in the previous round have been addressed, and I have verified each against the revised manuscript rather than the response letter alone.

**********

what does this mean?). If published, this will include your full peer review and any attached files.

**Do you want your identity to be public for this peer review?**  If you choose “no”, your identity will remain anonymous but your review may still be made public.

For information about this choice, including consent withdrawal, please see our Privacy Policy

Reviewer #1: **Yes:**  Ismaël Maatouk

**********
